# Continued use of retracted papers: Temporal trends in citations and (lack of) awareness of retractions shown in citation contexts in biomedicine

**DOI:** 10.1162/qss_a_00155

**Published:** 2022-02-04

**Authors:** Tzu-Kun Hsiao, Jodi Schneider

**Affiliations:** School of Information Sciences, University of Illinois at Urbana-Champaign, Champaign IL, USA

**Keywords:** citation analysis, citation context analysis, intentional postretraction citation, postretraction citation PubMed Central Open Access Subset, retraction

## Abstract

We present the first database-wide study on the citation contexts of retracted papers, which covers 7,813 retracted papers indexed in PubMed, 169,434 citations collected from iCite, and 48,134 citation contexts identified from the XML version of the PubMed Central Open Access Subset. Compared with previous citation studies that focused on comparing citation counts using two time frames (i.e., preretraction and postretraction), our analyses show the longitudinal trends of citations to retracted papers in the past 60 years (1960–2020). Our temporal analyses show that retracted papers continued to be cited, but that old retracted papers stopped being cited as time progressed. Analysis of the text progression of pre- and postretraction citation contexts shows that retraction did not change the way the retracted papers were cited. Furthermore, among the 13,252 postretraction citation contexts, only 722 (5.4%) citation contexts acknowledged the retraction. In these 722 citation contexts, the retracted papers were most commonly cited as related work or as an example of problematic science. Our findings deepen the understanding of why retraction does not stop citation and demonstrate that the vast majority of postretraction citations in biomedicine do not document the retraction.

## PEER REVIEW

https://publons.com/publon/10.1162/qss_a_00155

## 1. INTRODUCTION

Although retraction is intended to remove published research from the citable literature, retraction does not stop the diffusion of the retracted paper. Empirical studies have shown that papers continue to be cited after being retracted (Bar-Ilan & Halevi, 2017; Bolland, Grey, & Avenell, 2021; Candal-Pedreira, Ruano-Ravina et al., 2020; Dal-Ré & Ayuso, 2020; Mott, Fairhurst, & Torgerson, 2019; Pfeifer & Snodgrass, 1990; Theis-Mahon & Bakker, 2020; van der Vet & Nijveen, 2016). For example, Dal-Ré and Ayuso (2020) studied citations to 460 retracted genetics articles and found that 23% of the citations were postretraction citations.

Although citations to retracted papers have been widely discussed in previous studies, the bulk of studies focused on a small number of retracted papers (e.g., 15 retracted papers in Bar-Ilan and Halevi [2017]) or a single retracted paper (Schneider, Ye et al., 2020; Suelzer, Deal et al., 2019; van der Vet & Nijveen, 2016). Only citation counts and citation networks have been studied at scale. However, many citation studies focused on a particular field (e.g., retracted articles in genetics (Dal-Ré & Ayuso, 2020), in dentistry (Theis-Mahon & Bakker, 2020), and in engineering (Rubbo, Pilatti, & Picinin, 2019)). A few analyses on retracted papers in one database or in the intersection of multiple databases reported that retracted papers and their authors’ citation counts significantly decreased after the retraction (Dinh, Sarol et al., 2019; Shuai, Rollins et al., 2017). However, postretraction citations can persist for more than 10 years (Kim, Yi et al., 2019; Schneider et al., 2020), and retracted papers may be deeply embedded in citation networks (Chen, Hu et al., 2013). Prior work showed that although retracted papers and their authors were penalized with fewer citations, the retracted papers still circulated in scientific communities.

Studies of citation counts and citation networks have been limited to showing the existence and quantity of postretraction citations: How retracted papers were cited in the full-text articles was underexplored. Studies that explored full-text articles mainly focused on the acknowledgment of retraction shown in citation contexts (Budd, Coble, & Abritis, 2016; Neale, Dailey, & Abrams, 2010; Piller, 2021; Schneider et al., 2020; Suelzer et al., 2019) and the tone (positive, negative, and neutral) of each context (Bar-Ilan & Halevi, 2017; Hamilton, 2019; Theis-Mahon & Bakker, 2020; Yang, Qi, & Diao, 2020). Furthermore, to the best of our knowledge, Budd et al. (2016) is the only study covering citation contexts of retracted papers at scale, but they only examined the acknowledgment of retraction shown in citations to 265 papers in MEDLINE retracted between 2001 and 2005.

Our goal is to deepen the understanding of citations to retracted papers through large-scale analyses of citations and citation contexts. Our citation analyses investigate the temporal trends of citations to PubMed-indexed retracted papers. Compared with previous citation studies that focused on comparing citation counts using two time frames (i.e., preretraction and postretraction), our analyses show the longitudinal trends of citations to retracted papers in the past 60 years (1960–2020). In contrast to previous citation context analyses (which focused on the tones and the number of citation contexts acknowledging retraction), our analyses reveal the locations of the citation contexts mentioning retracted papers in full-text articles, contribute to the understanding of acknowledgment of retraction shown in citation contexts at scale, and indicate the purposes for intentionally citing retracted papers. In particular, we analyze citation contexts extracted from PubMed Central (PMC) open access articles that cited the retracted papers. The present work is the first database-wise study to examine citation contexts of retracted papers without time limitations.

In this study, we aim to answer the following research questions (RQs):

**RQ1:** How were PubMed-indexed retracted papers cited over time?
**RQ2:** How were papers cited in full-text articles before and after their retractions?
**RQ3:** How many postretraction citations acknowledged the retractions?
**RQ4:** What were the purposes for intentionally citing retracted papers?

We used two sets of data to answer the RQs. For RQ1, we investigated iCite citations to retracted papers indexed in PubMed. For RQ2, RQ3, and RQ4, we analyzed retracted papers in PubMed and citation contexts extracted from PMC open access articles. Our citation analyses deepen our understanding of the citation patterns of retracted biomedical research papers by tracing citations to retracted papers in the past 60 years. Our investigation of citation contexts to a vast collection of retracted papers reveals the locations where retracted papers were cited in full-text articles before and after their retractions and the purposes for intentionally citing retracted papers.

## 2. LITERATURE REVIEW

### 2.1. Citations to Retracted Papers

Citations to retracted papers have been studied since the 1990s (Budd, Sievert et al., 1999; Kochan & Budd, 1992; Pfeifer & Snodgrass, 1990; Wright, 1991). However, large-scale studies are few in number. Budd et al. (1999) analyzed retracted papers found in the MEDLINE database. They found 235 retracted papers and identified 2,034 postretraction citations in the Science Citation Index. Budd, Coble, and Anderson (2011) continued this line of research by studying 1,112 retracted papers in PubMed published from 1997–2009 and examined citations (obtained from Scopus) to papers retracted in 2000 and 2005. In the 2000 set, four retracted papers had no postretraction citation, and 14 (77.78%) out of the 18 retracted papers received 325 postretraction citations. In the 2005 set, 68 retracted papers received 965 postretraction citations. Dinh et al. (2019) collected a set of biomedical retracted papers from the intersection of PubMed, Scopus, and Retraction Watch. Their results showed that postretraction citation counts were significantly lower than preretraction citation counts (Dinh et al., 2019). Moreover, for 250 retracted articles with the same preretraction and postretraction time frame, citation counts decreased in the postretraction timeframe for 96% (240/250) of the retracted articles (Dinh et al., 2019). Chen et al. (2013) and Shuai et al. (2017) studied retracted papers indexed in Web of Science (WoS). Chen et al. (2013) used cocitation networks to visualize how retracted papers were cited. Shuai et al. (2017) reported that citation counts of retracted papers and their authors significantly decreased after retraction.

In contrast to large-scale studies, citation analyses of retracted papers in a particular field are abundant (Bolboacă, Buhai et al., 2019; Dal-Ré & Ayuso, 2020; Hamilton, 2019; Madhugiri, Nagella, & Uppar, 2021; Pantziarka & Meheus, 2019; Rubbo et al., 2019; Theis-Mahon & Bakker, 2020; Yang et al., 2020). For example, Madhugiri et al. (2021) examined citations to 191 retracted papers in clinical neurosurgery as well as in allied clinical and basic science specialties. They reported that postretraction citations accounted for 50% of all the citations received by the retracted papers (Madhugiri et al., 2021). Rubbo et al. (2019) and Theis-Mahon and Bakker (2020) studied 238 retracted engineering articles and 136 retracted dentistry articles, respectively. Interestingly, they reported similar rates of retracted papers having postretraction citations. In particular, 156 (65.55%) out of the 238 retracted engineering articles and 84 (61.76%) out of the 136 retracted dentistry articles were cited after the retraction. Pantziarka and Meheus (2019) reported that the retracted oncology papers received 35.1 citations on average. Mott et al. (2019) studied citations to 387 retracted randomized controlled trials (RCTs). They reported that the retracted RCTs continued to be cited, yet the citations decreased after retraction. Candal-Pedreira et al. (2020) compared the pre- and postretraction citations to 304 research articles and literature reviews retracted between 2014 and 2016. They reported that a decrease in the number of citations was only observed in retracted papers published in first-quartile journals in Journal Citation Report.

Another set of citation analyses investigated retracted papers by one or a few authors (Bolland et al., 2021; Hagberg, 2020; Kochan & Budd, 1992), or a single retracted paper (Schneider et al., 2020; Suelzer et al., 2019; van der Vet & Nijveen, 2016). Bolland et al. (2021) examined citations to papers by two authors^1^ having integrity concerns and reported that 237 (6%) of 3,925 citations were made after the publication of either a retraction notice or an expression of concern. Van der Vet and Nijveen (2016) visualized the growth of the citation network linking to a retracted paper published in *Nature*. Schneider et al. (2020) explored the possible spread of information in a retracted paper through two generations of citations. They examined 35 out of 44 direct postretraction citations describing the retracted paper’s methods or results and found 161 second-generation citations citing these 35 papers. Furthermore, in the 161 second-generation citations, 23 citations relied on the information from the retracted paper without directly citing it (Schneider et al., 2020). Although both studies are case studies of a single retracted paper, their findings have raised the concern that information in the retracted papers might be disseminated through indirect chains of citations. As addressed in van der Vet and Nijveen (2016), proper citing behavior may amend the spread of retracted results, highlighting the need to study how retracted papers are cited in full-text articles at scale.

### 2.2. Citation Contexts Citing Retracted Papers

Citation context analysis has been used to explore authors’ motivations, purposes, and intents for citing papers (Jha, Jbara et al., 2017; Teufel, Siddharthan, & Tidhar, 2006). Both the locations of citations in full-text articles and the text surrounding the citations have been studied (Bertin, Atanassova et al., 2016; Ding, Zhang et al., 2014; Tahamtan & Bornmann, 2019). For example, Bertin et al. (2016) reported that the density of citations appearing in full text is related to the citing paper’s structure (i.e., the IMRaD sections: introduction, method, result, and discussion). They reported that citations mostly appeared in the introduction and discussion sections. Studies on the text surrounding citations have shed light on the semantics of citations and citation motives. For instance, papers are rarely cited in a negative tone (Jha et al., 2017; Teufel et al., 2006). Moreover, citations can be made for various reasons, such as *showing related work*, *use*, *comparison*, and *corroboration* (Garfield, 1965; Hernández-Alvarez, Soriano, & Martínez-Barco, 2017; Jha et al., 2017; Li, He et al., 2013; Valenzuela, Ha, & Etzioni, 2015). Garfield (1965) first proposed a scheme containing 15 reasons for citing articles. Agarwal, Choubey, and Yu (2010) classified 1,710 citation contexts in 43 open-access biomedical articles into eight categories: *background*/*perfunctory*, *contemporary*, *contrast*/*conflict*, *evaluation*, *explanation of results*, *material*/*method*, *modality*, and *similarity*/*consistency*.

However, citation contexts citing retracted papers are underexplored. Existing studies have only investigated whether the citation contexts acknowledged the retractions, and not why. For instance, in a case study following a single retracted paper published in *Nature*, van der Vet and Nijveen (2016) found that, among the 57 citations given after the year of retraction, only two postretraction citations (3.5%) showed awareness of the retraction in the text. Another case study by Schneider et al. (2020) showed that, in 112 postretraction citations to a retracted paper, only five citations (4.5%) mentioned the retraction. Budd et al. (2016) examined citations to 265 retracted papers index in MEDLINE using a time window between 2001 and 2005. Only 4.15% (204 out of 4,917 citations) acknowledged the retraction (Budd et al., 2016). Table 1 summarizes the percentage and number of citations acknowledging retraction as reported in previous research.

**Table 1.** Citations acknowledging retraction reported in previous research

Reference	% citations acknowledging retraction	# citations acknowledging retraction in the citations included for analysis	# retracted papers included for citation analysis
Santos-d’Amorim, de Melo, and dos Santos (2021)	37.85	81/214 citations	One retracted Covid-19 paper
Piller (2021)	47.5	95/200 citations	Two retracted Covid-19 papers
Van der Walt, Willems et al. (2020)	21.05	4/19 sampled citations	33 retracted Covid-19 papers
Schneider et al. (2020)	4.5	5/112 citations	One retracted paper
Theis-Mahon and Bakker (2020)	5.4	37/685 citations	81 retracted dentistry papers
Yang et al. (2020)	16.03	21/131 citations	46 retracted psychological papers
Bolboacă, Buhai et al. (2019)	1.07	6/559 citations	54 retracted papers reporting a radiology-imaging diagnostic method
Hamilton (2019)	6.6	27/407 citations	47 retracted radiation oncology papers
Suelzer et al. (2019)	38.2 (2005–2010); 71.7 (2011–2018)	123/322 (2005–2010); 360/502 (2011–2018)	Wakefield’s *Lancet* paper (partly retracted in 2004; fully retracted in 2010)
Bornemann-Cimenti, Szilagyi, and Sandner-Kiesling (2016)	25.8	–/267 citations^a^	20 retracted papers by Scott S. Reuben
Budd et al. (2016)	4.15	204/4,917 citations	265 retracted papers in MEDLINE
van der Vet and Nijveen (2016)	3.5^b^	0/37 citations in 2014; 2/57 citations in 2015	A paper published in *Nature* and retracted in Feb. 2014.
Budd et al. (2011)	6	14/247 citations in the 2000 sample; 8/144 citations in the 2005 sample	1,112 retracted papers in PubMed
Neale et al. (2010)	2.8	17/603 citations stratified random sampled from 5,393 citations	102 papers affected by scientific misconduct
Redman, Yarandi, and Merz (2008)	< 3 in 9/10 papers; 29 in the paper having 96 citations	–/225 citations^a^ to 10 papers with high postretraction citation rates (citation per-paper range from 10–96)	315 retracted papers in PubMed
Budd et al. (1999)	6.4 (AIM); 7.7 (non-AIM)	19/299 citations from AIM journals; 123/1,594 citations from non-AIM journals	235 retracted papers in MEDLINE
Kochan and Budd (1992)	5.7	17/298 citations	John Darsee’s papers
Pfeifer and Snodgrass (1990)	2.9	5/178 citations	82 retracted papers identified from journals in *Index Medicus*

^a^ Number not reported.

^b^ Inferred from data: 2/57 citations in 2015.

As observed in the previous studies (Table 1), papers continued to be cited after being retracted, and the citing papers rarely informed readers about the retractions. Five out of nine previous studies published in the last 5 years (2016–2021) reported fewer than 7% of citations acknowledging retractions (Bolboacă et al., 2019; Budd et al., 2016; Hamilton, 2019; Schneider et al., 2020; van der Vet & Nijveen, 2016). Interestingly, studies on infamous, high-profile retracted papers (Bornemann-Cimenti et al., 2016; Piller, 2021; Suelzer et al., 2019) found higher rates of citations acknowledging retractions.

Some studies examined the tone (positive, negative, and neutral) of each citation context (Bar-Ilan & Halevi, 2017; Kochan & Budd, 1992; Theis-Mahon & Bakker, 2020; Yang et al., 2020). Bar-Ilan and Halevi (2017) described how 15 retracted articles were mentioned in 238 citing documents after being retracted. They discovered that the retracted papers were mostly mentioned in a positive tone and that citing papers rarely mentioned that the cited article had been retracted. Yang et al. (2020) studied 131 postretraction citations to 46 retracted psychology papers and found 119 (90.84%) citations were positive. Theis-Mahon and Bakker (2020) reported that 475 (69.34%) out of the 685 postretraction citations to retracted dentistry papers were positive. Santos-d’Amorim et al. (2021) investigated 214 postretraction citations to one retracted Covid-19 paper and identified 64 (30%) positive, 81 (38%) negative, and 69 (32%) neutral citations. Studies that more deeply analyzed citation contexts (e.g., beyond the tone) focused only on a single retracted paper and its citations (Fulton, Coates et al., 2015; Schneider et al., 2020; Suelzer et al., 2019; van der Vet & Nijveen, 2016). The most fine-grained categorization of the citation contexts was proposed by Suelzer et al. (2019). They used eight categories (*affirmative*, *assumptive*, *conceptual*, *contrastive*, *methodological*, *negative*, *perfunctory*, and *persuasive*) to annotate 1,153 citations to Wakefield’s infamous *Lancet* paper connecting the MMR vaccine to autism (Suelzer et al., 2019).

By contrast, the present work focuses on the temporal trends of citations to retracted papers and examines citation contexts to a vast collection of retracted papers. We analyze how retracted papers in PubMed were cited over time and investigate the appearance of citation contexts mentioning these retracted papers in full-text articles. Different from previous citation studies comparing citation counts using two time frames (i.e., preretraction and postretraction), our citation analyses present the longitudinal trends of citations to retracted papers in the past 60 years (1960–2020). Going beyond the tones of citation contexts, we examine where retracted papers were mentioned in full-text articles and identify the purposes for intentionally citing retracted papers. Our approach of systematically identifying citations acknowledging retraction in PMC open access articles reveals acknowledgment of retraction shown in citation contexts at scale.

## 3. METHODS

### 3.1. Data

The data for this study was collected from various sources. In particular, the retracted papers were collected from PubMed, the citations to the retracted papers were collected from iCite, and the citation contexts were identified from the PMCOA Citation Context Dataset (Hsiao & Torvik, manuscript in preparation), which contains citation contexts identified from over two million PMC open access articles. The following sections describe how the retracted papers, citations to retracted papers, and citation contexts of retracted papers were collected. The data set used for this study has been deposited to the Illinois Data Bank (Hsiao & Schneider, 2021).

#### 3.1.1. Retracted papers indexed in PubMed

Retracted papers in PubMed were searched using the query “*retracted publication*” [*PT*] on August 20, 2020. The search yielded 7,813 results, including four retraction notices incorrectly indexed as retracted papers (Supplementary material 1). We corrected our data set to use the retracted papers associated with these four retraction notices, based on their titles and PubMed’s *retraction in* links. Using PubMed’s *retraction in* information, the retraction years of 7,766/7,813 (99.40%) retracted papers were identified.

#### 3.1.2. Citations to retracted papers

Citations to the retracted papers were collected on August 20, 2020. The citation data were collected from iCite^2^, which provides an API for accessing citation data from the NIH Open Citation Collection (NIH-OCC). The NIH-OCC contains citation data from PubMed, PMC, MEDLINE, and CrossRef (Hutchins, Baker et al., 2019). We collected 171,537 citations to retracted papers. However, 2,088 citations were from retraction notices (citing 1,970 retracted papers) and 15 citations were problematic because the citing and the cited retracted papers have the same PMID. In the 1,970 retracted papers cited by retraction notices, 1,719 retracted papers had citations from articles other than retraction notices, and 251 retracted papers were only cited by retraction notices. After excluding the 2,088 citations from retraction notices and the 15 problematic citations, 6,704 (85.81%) retracted papers were cited 169,434 times, and 1,109 (14.19%) retracted papers had never been cited.

Different approaches have been used to identify postretraction citations, such as after 1 month (Mott et al., 2019), after 6 months (Hamilton, 2019), and after the calendar year of retraction (Budd et al., 2011, 2016; Madhugiri et al., 2021; Pfeifer & Snodgrass, 1990). Here we operationalize a postretraction citation as a citation made after the calendar year of retraction. This follows the most similar studies of retracted papers in PubMed (Budd et al., 2011, 2016), which, like our study in PubMed Central, focused on biomedicine/medicine. Also, we expect this time frame to allow retraction notices to have been apparent to the citing authors at manuscript submission, on average, because previous research has reported mean submission-to-publication time as 120 days for biomedical journals published by *Nature* Publishing Group and 139 days for BioMed Central journals (Dong, Loh, & Mondry, 2006). Similarly, medical journals have median submission-to-publication time of 224 days (min 24 days; max 1,034 days) (Sebo, Fournier et al., 2019).

Table 2 presents the number of citations to retracted papers made in three intervals: before the year of retraction (preretraction), in the year of retraction, and after the year of retraction (postretraction). As shown in Table 2, 75% of the retracted papers were cited no more than six times after retraction.

**Table 2.** Descriptive statistics of the number of citations

	Preretraction	In retraction year	Postretraction	Total
#Retracted papers	7,766^a^	7,766^a^	7,766^a^	7,813
#Citations^b^				
Mean	12.86	2.74	6.20	21.69
SD	40.28	7.23	17.87	55.25
Q1	0.00	0.00	0.00	2.00
Q2	1.00	1.00	2.00	7.00
Q3	10.00	3.00	6.00	21.00
Max	1440.00	299.00	844.00	2011.00

^a^ 47 papers lacked retraction year information.

^b^ 2,088 citations from retraction notices and 15 problematic citations have been excluded.

#### 3.1.3. Citation contexts of retracted papers

We identified citation contexts citing retracted papers using the PMCOA Citation Context Dataset (Hsiao & Torvik, manuscript in preparation), which is built from a snapshot of the XML version of PMC open access articles (PubMed Central, n.d.) retrieved in May 2019^3^. The PMCOA Citation Context Dataset contains citation contexts identified from 2,049,871 articles in the XML version of the PMC Open Access Subset (Hsiao & Torvik, manuscript in preparation). The citation contexts were identified leveraging XML tags in the Journal Publishing Tag Set (JATS), the standardized tagging guideline used by PMC. In particular, <ref> tags and <xref> tags were used to identify the references and the cross-references to the objects within the document, respectively. The appearance of citations in the full-text articles was identified by mapping the IDs associating with the <ref> tags and <xref> tags. For each article, after identifying citations in the document, the article was parsed into sentences. Further details of the method for identifying the citation contexts are described in Hsiao and Torvik (manuscript in preparation).

In the PMCOA Citation Context Dataset (Hsiao & Torvik, manuscript in preparation), each citation context is a sentence (or a text cell in a table) containing at least one citation. For each citation context, the data set has the following information: PMID and PMCID of the citing paper, ID and PMID (if applicable) of the cited paper, the citation context’s location (abstract, main text, supporting material, and table/figure caption), the IMRaD section (introduction/background, method, results, and conclusion/discussion), and text progression. We identified the citation contexts citing retracted papers from the PMCOA Citation Context Dataset by mapping the cited papers’ PMIDs to the retracted papers’ PMIDs.

Of the 48,747 citation contexts citing retracted papers, we excluded 612 contexts that were from retraction notices, and one problematic citation context where the citing and cited retracted paper have the same PMID. The remaining 48,134 citation contexts were analyzed. As shown in Table 3, within the 48,134 citation contexts citing retracted papers, 13,252 (27.53%) were postretraction citation contexts. In these 13,252 postretraction citation contexts (i.e., citations made after the calendar year of retraction), 2,763 out of 7,813 (35.36%) retracted papers were cited.

**Table 3.** Citation contexts of citations to retracted papers

	Preretraction (%)		In retraction year (%)		Postretraction (%)		Missing retraction year (%)	
Total	28,439	(100.00)	6,412	(100.00)	13,252	(100.00)	31	(100.00)
In main text								
In introduction/background	6,952	(24.45)	1,698	(26.48)	3,947	(29.78)	17	(54.84)
In methods	2,071	(7.28)	388	(6.05)	679	(5.12)	2	(6.45)
In results	3,249	(11.42)	708	(11.04)	1,190	(8.98)	1	(3.23)
In conclusion/discussion	8,089	(28.44)	1,881	(29.34)	4,156	(31.36)	4	(12.90)
IMRaD not identified^a^	6,883	(24.20)	1,414	(22.05)	2,764	(20.86)	6	(19.35)
In abstract	14	(0.05)	4	(0.06)	1	(0.01)	0	(0.00)
In supporting material	4	(0.01)	6	(0.09)	5	(0.04)	0	(0.00)
In tables and table/figure captions	1,177	(4.14)	313	(4.88)	510	(3.85)	1	(3.23)

^a^ Sections for which the IMRaD section types were unidentifiable through the method described in Hsiao and Torvik (2020).

### 3.2. Evaluating the Citedness of Retracted Papers over Time

The citation data collected from iCite (as described in Section 3.1.2) were used to evaluated the citedness of retracted papers over time. The retracted papers were categorized into *active*, *inactive*, and *uncited*. We define these terms as follows:

1. *Active*: Papers actively receiving citations until a given year or beyond.
2. *Inactive*: Papers having been cited in the past but no longer being cited in a given year and beyond.
3. *Uncited*: Papers never having been cited as of a given year.

For instance, PMCID:2226778 was cited in 1991–1993, 1995, 1998, and 2002. Hence, this paper was active until 2002 and became inactive in 2003 because the last citation appeared in 2002.

### 3.3. Identifying Intentional Postretraction Citations

From the 13,252 postretraction citation contexts described in Section 3.1.3, we identified the postretraction citations that intentionally cited retracted papers, using three rules (Table 4). The first two rules captured possible intentional postretraction citations via cue words that possibly referred to retractions. In particular, the first rule identified whether at least one of the cue words appeared in the citation context. The second rule identified whether at least one of the cue words appeared in the acknowledgment window, which we defined as the five sentences before or after the citation context. The third rule captured the condition when the retracted paper and the retraction notice were cited together. As the cue word approach might falsely capture some citations that did not refer to the retractions, we manually reviewed each identified citation context. Our manual review adjusted for the drawback of using cue words and ensured that each identified citation context was an intentional postretraction citation.

**Table 4.** Postretraction citation contexts acknowledging the retraction

Priority	Rule	# citation contexts identified	# citation contexts acknowledging the retraction	# false positives*
1	At least one of the cue words (retract*, withdr*, and error) appears in the citation context	243	169	74
2	At least one of the cue words (retract*, withdr*) appears in the acknowledgment window	309	283	26
3	Retraction notice is cited together with the retracted paper in the citing paper’s full text	159	159	0
Total		711	611	100

* Cue words do not always refer to retraction. We manually inspected the identified citation contexts to exclude false positives. Some examples: retract* in *neurite retraction*; withdr* in *withdrawal symptoms*; *error* in *error rate*.

Note that a retracted paper may be mentioned multiple times in the citing paper’s full text (i.e., having multiple citation contexts in the citing paper). When there are multiple citation contexts, the acknowledgment of retraction may only appear in one of the citation contexts. We counted all the citation contexts in a paper as acknowledging the retraction if at least one did so explicitly. There were 611 postretraction citation contexts explicitly acknowledging the retraction (identified by the rules in Table 4). Using the PMCIDs of the citing papers and the PMIDs of the cited retracted papers of these 611 postretraction citation contexts, we further identified 111 implicit intentional postretraction citation contexts. In sum, we identified 722 intentional postretraction citation contexts in 430 papers.

Table 5 shows the locations of these 722 intentional postretraction citation contexts. Of these, 685 (94.88%) were found in the main text, and 34 (4.71%) were found in tables, table captions, or figure captions. For the citation contexts in the main text, 284 (39.34%) citation contexts did not have IMRaD sections identified. Of the sections where we identified IMRaD section types, the introduction/background sections had the largest share (21.33%) of citation contexts acknowledging retraction, followed by the discussion/conclusion sections (15.24%).

**Table 5.** Locations of postretraction citation contexts

	# postretraction citation contexts acknowledging retraction	(%)
In main text		
In introduction/background	154	(21.33)
In methods	32	(4.43)
In results	105	(14.54)
In discussion/conclusion	110	(15.24)
IMRaD not identified	284	(39.34)
In supporting material	3	(0.42)
In tables and table/figure captions	34	(4.71)
Total	722	(100)

### 3.4. Identifying Purposes for Intentionally Citing Retracted Papers

To understand citing authors’ purposes for intentionally citing retracted papers, we manually inspected the text of each citation context. For each citation context, we first read the citing article’s title, the section title for the section in which the citation context appeared, and the paragraph containing the citation context. If the citation purpose could not be discerned even after reading the paragraph, we read the abstract and skim-read the paper to identify the purpose. Furthermore, a decision map (Supplementary material 2) was used to aid in choosing the most appropriate citation purpose.

For annotating the citation purposes, we created a classification scheme consisting of 11 categories. Table 6 presents the description of each category (see Supplementary material 3 for the example of each category). Aside from the citation purposes commonly reported in previous studies (e.g., *Related work*, *Comparison*, and *Use*), we also observed purposes relating to the unique nature of our data set: citations to retracted work. These particular purposes are *Republication of retraction*, *Example of problematic science*, and *Notify retraction included*.

**Table 6.** The citation purposes

Purpose	Description
Comparison	Authors of the citing paper compared “their” results or methods with the retracted paper. According to the tone, this category is further divided into negative (−), positive (+), and neutral (±). Negative tone refers to the cases that inconsistency, contradiction, or discrepancy is reported in the comparison. Positive tone refers to the cases that consistency is reported in the comparison. Neutral tone refers to the cases where consistency between the compared results was unclear.
Correction	The retracted paper was cited to make a correction.
Example of problematic science	The retracted paper was cited to provide an example of problematic science. This purpose satisfies one of the following conditions: (a) the retracted paper was cited to provide an example of problematic research (e.g., irreproducible research, unreliable research, research involving scientific misconduct, a flawed study); (b) the retracted paper was cited to provide an example of where peer review failed and problematic science was published; (c) the retracted paper was cited to provide an example showing a problem in scientific research or scholarly communication; or (d) the retracted paper was cited to provide an example of the societal impact of problematic research.
Exclusion rationale	The retracted paper was cited to explain why it is excluded from use/consideration. Especially found in the context of research synthesis (e.g., review articles and meta-analyses that provide a formal exclusion rationale for papers that are not included.) This purpose can also be found in the literature review section of a research article.
Notify retraction included	Notify readers that one or more retracted papers were included in a different, previous published review article, guideline, or paper.
Related work	The retracted paper was cited to show what has been done or found in the past or was cited for one of the following reasons: (a) The retracted paper was once a landmark in the field; (b) the retracted paper was the origin/pioneer of something (e.g., “X first identified/describe Y”, “X was identified as a novel …”, “X was initially proposed…”, or “X was originally…”); and (c) the retracted paper led to an important event in the field, such as Wakefield’s paper’s influence on the supposed autism–vaccine link and the antivaccine movement.
Republication of retraction	In the republication of the retracted paper, the authors cited the retracted paper to announce the republication.
Reproduce	A citation to the retracted paper was made because the citing paper tried to reproduce/repeat the finding or experiment mentioned in the retracted paper.
Subject of study	Cited retraction is the object of study of a case study about retraction, or is the data used in a study about retraction, scientific misconduct, or peer review. Note that in these studies, retracted papers can be cited in the results.
Use	Citing paper uses something from the cited retracted paper. This type of citation is often found in the Methods section.
Other	Those that do not belong to the above categories.

## 4. RESULTS

### 4.1. Years between Publication and Retraction

**Figure 1.**
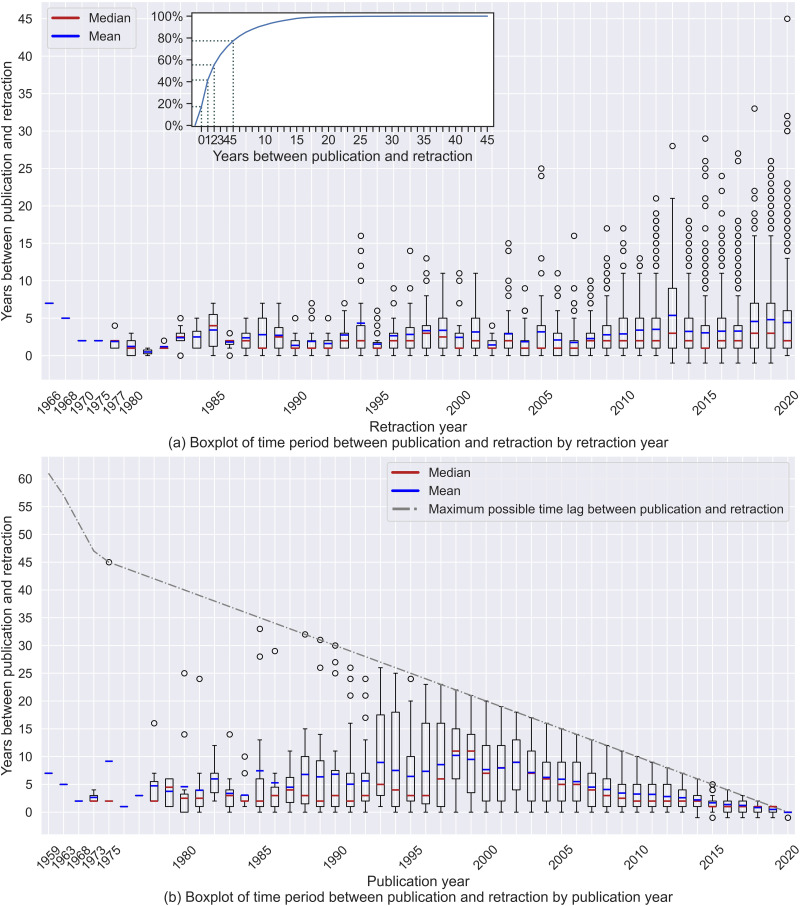
Boxplot of time lag between publication and retraction arranged by retraction year and publication year. The box areas show interquartile range (IQR, from 25% to 75%) of the time lag between publication and retraction. The upper whisker of each box is the longest time lag smaller than 1.5 IQR above the 75th percentile; the lower whisker is the shortest time lag greater than 1.5 IQR below the 25th percentile. The grey dotted line in the lower panel shows the maximum possible time lag (i.e., up to 2020, the year of data collection) for papers to be retracted in each publication year.

Figure 1 shows a boxplot of the number of years between publication and retraction arranged by retraction year and publication year (using the data described in Section 3.1.1). Note that 47 of our 7,813 retracted papers lack a retraction year in PubMed^4^, so this figure is built on the remaining 7,766 retractions. The first retracted paper (PMID: 13850774) was published in 1959 and was retracted in 1966. Eleven papers were retracted 1 year *before* their publication year; these papers might have been retracted in the *online-first* stage. Also, 4,308 (55.47%) papers were retracted within 2 years after publication. Figure 1(a) shows that for each year after 1986, the distribution of the time period between publication and retraction was right skewed, indicating that the majority of retractions happened within a short time after publication. Combining the distribution with the medians, the graph shows that at least 50% of the retractions happened no more than 3 years after publication, regardless of the retraction year.

Figure 1(b) also shows the number of years between publication and retraction but is arranged by publication year. This graph shows that retraction is an ongoing process. In calendar year 2020, at least one paper was retracted from each publication year from 1997–2020. Furthermore, publishers continued to retract papers published a long time ago. For example, in 2020, a 45-year-old paper published in 1975 was retracted. The continued retraction of old publications highlights that papers that are not currently retracted may be retracted in the future. The graph also reveals the right-censored nature of the data set: Retractions typically happen after publication; hence, the more recently a paper was published, the shorter the possible time between publication and retraction.

### 4.2. Temporal Trends of Citations to Retracted Papers

**Figure 2.**
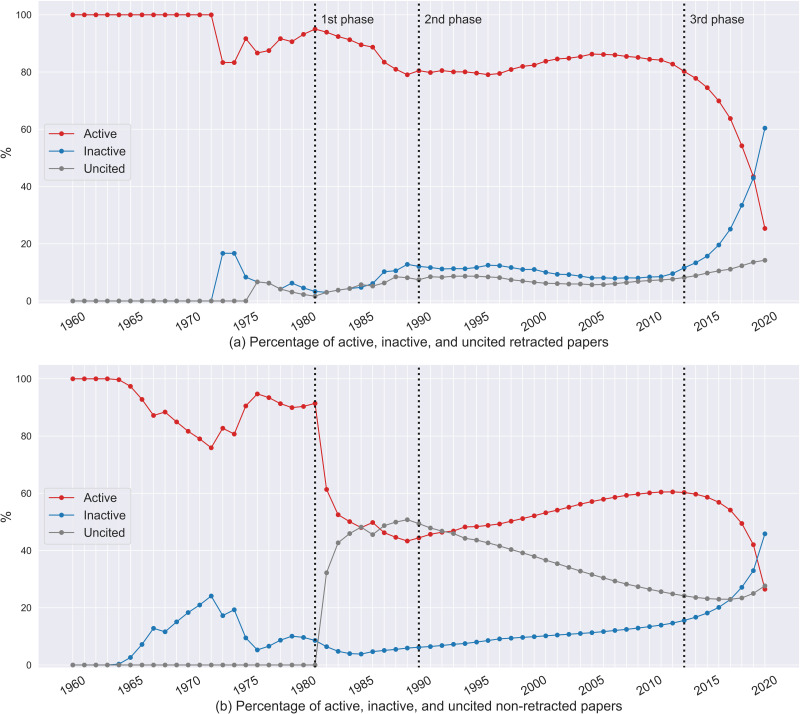
Distribution of active, inactive, and uncited papers.

This section analyzes citations to retracted papers (using the data described in Section 3.1.2). Figure 2 shows the citedness of retracted papers and nonretracted papers over time. To see whether retracted and nonretracted papers show similar trends, we collected nonretracted papers from the August 2020 iCite database snapshot (iCite, Hutchins, & Santangelo, 2020). The cohort of nonretracted papers to compare to the retracted papers was selected in two steps: identifying nonretracted papers published in the same years as the retracted papers; and, for each publication year, selecting all nonretracted papers with the same citation counts as the retracted papers published in the same year.

As shown in Figure 2(a), the first uncited retracted paper was from 1976. This paper (PMID: 183981) was published in 1976 and retracted in 1977. We observed three phases (1981–1989, 1990–2012, and 2013–2020) in the growth and decline of *active* and *inactive* retracted papers. Since 1981, the proportion of *uncited* retracted papers gradually increased from 1.67% to 14.23%. In the first phase (1981–1989, to the left of the first line on Figure 2(a)), the proportion of *active* retracted papers decreased from 95% to 79.07%. In the second phase (1990–2012, middle of Figure 2(a)), the proportion of *active* retracted papers remained between 79.09% and 86.28%. Since 2013, the proportion of *active* retracted papers has dramatically decreased from 80.23% to 25.36% (right of Figure 2(a)). However, up to 2018, more than 50% of the retracted papers were active. Overall, these results show that retracted papers continued to be cited, but that the proportion of *active* retracted papers has decreased in recent years.

The decreasing trend in the proportion of *active* papers was also observed in nonretracted papers (Figure 2(b)) in the first phase and the third phase. In the first phase (1981–1989), the proportion of *active* nonretracted papers dropped from 91.42% to 43.32%. There was also a dramatic decrease of *active* nonretracted papers in the third phase (2013–2020), where the proportion of *active* nonretracted papers dropped from 60.28% to 26.49%. Together, the results show that the proportion of *active* papers has decreased in recent years, whether they were retracted or not.

**Figure 3.**
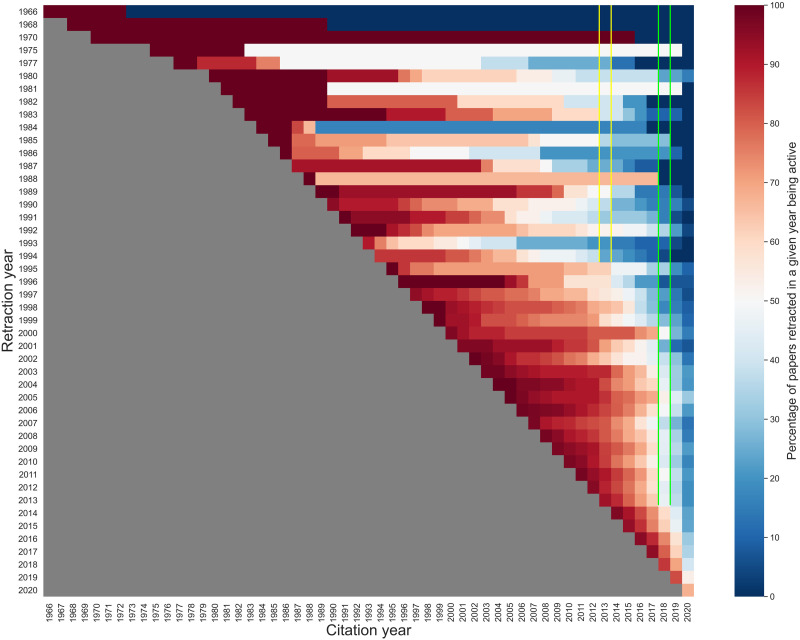
The percentage of active retracted papers with a given retraction year over time. Note that the citation data from 2020 are incomplete because the data were collected in August 2020. The shades denote the percentage of active retracted papers in a given citation year. Red shades denote that *more* than 50% of the papers retracted in a given year were active in a given citation year. Blue shades denote that *less* than 50% of the papers retracted in a given year were active in a given citation year. The grey area is the preretraction phase. The yellow box shows the percentages of papers retracted before 1995 that are active in citation year 2013. The green box shows the percentages of papers retracted before 2014 that are active in citation year 2018.

The decrease in *active* retracted papers could be partly explained by the fact that old retractions became inactive as time progressed (see Figure 3). Another possible effect is that citations take time to accumulate. Nane (2015) reported that although a paper was most likely to receive its first citation within two years after publication, the longest time window observed between publication and first citation was 13 years. Lachance and Larivière (2014) mentioned that for medicine and science papers, although most of the citations were received within five years after publication, a paper could be cited 30+ years after publication. Therefore, the growth of *uncited* and *inactive* retracted papers observed in the third phase (2013–2020) could be affected by the following reasons: For *uncited* retracted papers published in recent years, the time window might not have been long enough for receiving their first citations, or for *inactive* retracted papers published in recent years, there could be a gap between the year of last citation found in our data and future citations. In other words, *uncited* and *inactive* retracted papers could become *active* in the future.

Figure 3 further shows that older retracted papers stopped being cited as time progressed. In particular, Figure 3 is a visualization over time, showing the share of *active* retracted papers among the papers retracted in a given year. Note that, in our data set, no paper was retracted in 1967, 1969, 1971–1974, 1976, 1978, or 1979. Figure 3 further explains the dramatic decrease in the proportions of *active* retracted papers in the third phase (2013–2020, in Figure 2(a)). Across all retraction years, the percentage of *active* retracted papers decreased over time. In particular, in citation year 2013, for the papers retracted before 1995 (except the papers retracted in 1970 and 1988), the shares of *active* retracted papers were less than or about 50%; notice the blue shading of the area highlighted in the yellow box in Figure 3, whereas the remainder of the column below it is shaded in reds. Furthermore, in citation year 2018, the shares of *active* retracted papers were less than or about 50% among the papers retracted before 2014; notice the blue shading of the area highlighted in the green box in Figure 3, whereas the remainder of the column below it is shaded in reds. This echoes the dramatic decrease of active retracted papers observed in the third phase (2013–2020, in Figure 2(a)).

### 4.3. Characteristics of the Citation Contexts of Retracted Papers

**Figure 4.**
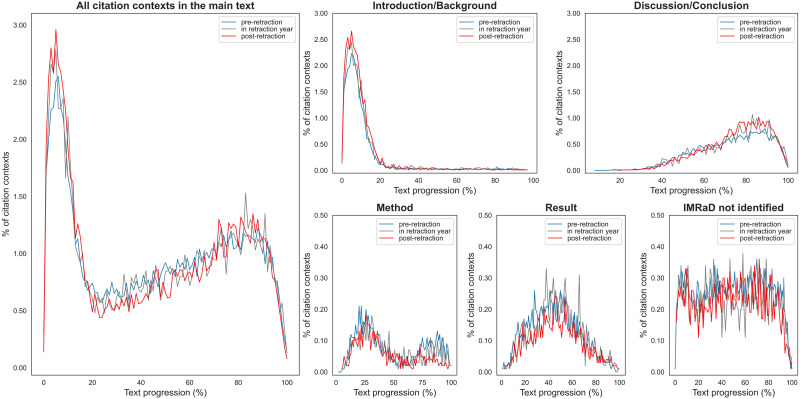
Locations of citations to retracted papers. Text progression indicates the location of a citation context in the *main text* on a percentage scale. The IMRaD sections were identified from the section titles (Hsiao & Torvik, 2020). *IMRaD not identified* refers to the sections where the IMRaD section types could not be identified from the section titles. The *y*-axis scales do not range from 0–100% because each part of the text only has a few citation contexts. For clarity, to show the trends of citation contexts’ locations the scales of the *y*-axes were set from 0–3% for all citation contexts as well as for citation contexts in introduction/background sections and discussion/conclusion sections. For citation contexts in method sections, in result sections, and in sections for which the IMRaD section types were unidentifiable, the scales of the *y*-axes were set from 0–0.5%.

As addressed in previous studies (Bertin et al., 2016; Dong & Schäfer, 2011) the location of citation implies the function of a cited work in the citing work. For instance, citations appearing in the introduction section may be cited to provide background knowledge, whereas citations appearing in the discussion/conclusion section may be used for making comparisons or supporting the reported findings. Figure 4 presents the locations of 46,069 citation contexts mentioning retracted papers found in the *main text* of PMC open access articles (as described in Section 3.1.3). Overall, the preretraction citation contexts and postretraction citation contexts show similar distributions of text progression. However, postretraction citation contexts were slightly more concentrated at the beginning and the end of papers. This implies that most retracted papers were cited for similar purposes both before and after they were retracted: Retraction did not change the way they were cited. The distribution of citation contexts in the IMRaD sections (introduction/background, method, results, and conclusions/discussion) revealed some further insights about how retracted papers were cited. In comparison to preretraction citations, more postretraction citations were found in the introduction/background and the discussion/conclusion sections.

To check whether citation patterns have changed over time, we first grouped citation contexts by age relative to retraction year and then by text progression. The age relative to retraction year is the difference between the citing paper’s publication year and the retraction year. For example, if a 2019 paper cited a paper retracted in 2017, the age relative to retraction year is 2. Hence, by definition, the ages relative to retraction of postretraction citations are positive; the ages relative to retraction of preretraction citations are negative; and the age relative to retraction of citations in the same year as the retraction is zero.

**Figure 5.**
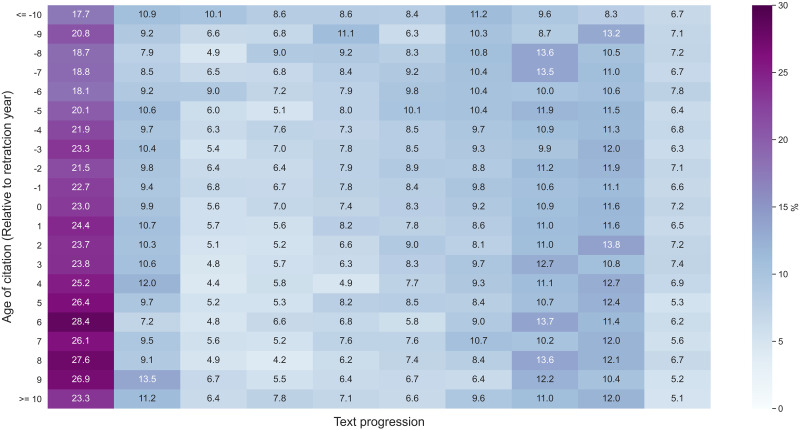
Distribution of text progression by the age of citation relative to retraction year. Age represents the number of years preceding (negative) or following (positive) retraction. Markers in the cells denote the percentage of citation context in each group of citation contexts having the same age relative to retraction year.

Figure 5 presents the text progression of citation contexts by age relative to retraction year. However, no distinct pattern was found. Citation contexts were mostly concentrated at the beginning of the papers, followed by the end of papers^5^, regardless of the age of citations relative to the retraction year.

Because a retracted paper can be mentioned multiple times in the full text of the citing paper, we further analyzed the number of mentions and the locations of citation contexts. Note that a paper may cite more than one retracted paper, and a retracted paper may be cited in multiple papers. Furthermore, each cited retracted paper may be mentioned one or multiple times in the citing paper’s full text. To capture how each citing paper mentions the cited retracted paper(s) in the full text, we pair citing papers and cited retracted papers as citing–cited pairs (i.e., a pair of a citing paper and a cited retracted paper). We found 30,283 citing–cited pairs in the 46,069 citation contexts mentioning retracted papers found in the *main text* of PMC open access articles. In these 30,283 citing–cited pairs, there were 16,895 preretraction pairs (55.79%), 3,983 pairs in the year of retraction (13.15%), and 9,405 postretraction pairs (31.06%). Of the 9,405 postretraction pairs, 422 pairs (4.49%) acknowledged retractions, and 8,983 pairs (95.51%) did not acknowledge retractions. In the following analyses, we omitted the pairs in the year of retraction for two reasons: The cited paper might have not been retracted when the citing author(s) submitted the manuscript; and The time frame might be too short to allow retraction notices to be apparent to the citing authors.

Table 7 shows the locations of retracted papers that are mentioned only once in the full text of the citing papers; 11,889 of the 16,895 preretraction pairs (70.37%) and 7,426 of the 9,405 postretraction pairs (78.96%) appeared only once. In the sections where IMRaD section types were identified, the mentions were mostly found in the introduction/background sections and the discussion/conclusion sections, regardless of preretraction or postretraction.

**Table 7.** Locations of the retracted papers being mentioned only once in the full text

IMRaD	# Preretraction pairs	(%)	# Postretraction pairs (retraction not acknowledged)	(%)	# Postretraction pairs (retraction acknowledged)	(%)
Introduction/background	3,272	(27.52)	2,359	(33.04)	47	(16.43)
Methods	690	(5.8)	397	(5.56)	13	(4.55)
Results	971	(8.17)	490	(6.86)	29	(10.14)
Discussion/conclusion	3,656	(30.75)	2,349	(32.90)	46	(16.08)
IMRaD not identified	3,300	(27.76)	1,545	(22.64)	151	(52.80)
Total	11,889	(100)	7,140	(100)	286	(100)

As for the retracted papers mentioned multiple times (hereafter referred to as multiple mentions), there were 5,006 preretraction pairs (29.63%) and 1,979 postretraction pairs (21.04%). Note that for a retracted paper with multiple mentions, all the mentions could appear in the same IMRaD section. As shown in Table 8, most of these multiple mentions were found in only one or two IMRaD sections. Pearson’s correlation analysis was performed to understand the relationship between the number of mentions and the number of IMRaD sections where the mentions appeared. For both pre- and postretraction pairs, the number of mentions was weakly correlated with the number of IMRaD sections where the mentions appeared (preretraction pairs: coef = 0.45, *p* < .001; postretraction pairs (retraction not acknowledged): coef = 0.29, *p* < .001; postretraction pairs (retraction acknowledged): coef = 0.47, *p* < .001). In other words, the higher the number of mentions, the more likely the multiple mentions would appear in more IMRaD sections.

**Table 8.** Number of different IMRaD sections where multiple mentions appeared

#Different IMRaD sections	# Preretraction pairs	(%)	# Postretraction pairs (retraction not acknowledged)	(%)	# Postretraction pairs (retraction acknowledged)	(%)
1	2,225	(44.45)	860	(46.66)	68	(50.00)
2	2,188	(43.71)	890	(48.29)	57	(41.91)
3	483	(9.65)	82	(4.45)	10	(7.35)
4	109	(2.18)	10	(0.54)	1	(0.74)
5	1	(0.02)	1	(0.05)	0	(0)
Total	5,006	(100)	1,843	(100)	136	(100)

**Figure 6.**
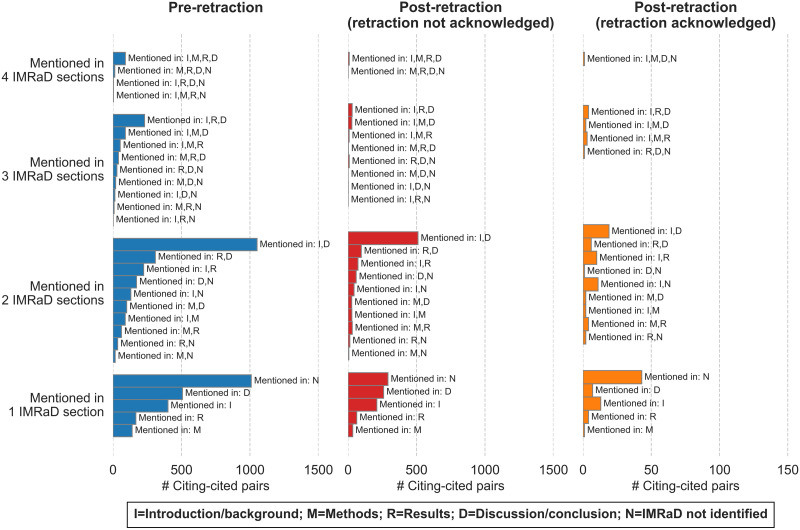
Location of the retracted papers that were mentioned multiple times.

Figure 6 shows the locations of the multiple mentions. Similar patterns were observed in preretraction pairs as well as in postretraction pairs that did not acknowledge the retraction. When the multiple mentions all appeared in one IMRaD section, the section was usually the discussion/conclusion, and second most common was the introduction/background. When the multiple mentions appeared in more than one IMRaD section, usually the combination was the introduction/background section along with the discussion/conclusion section. These similar patterns show that there was no substantial difference between how the retracted papers were cited before and after the retractions when the retractions were not acknowledged.

As for postretraction pairs with acknowledgment of retraction, the trends are slightly different. When the multiple mentions appeared in different IMRaD sections, these multiple mentions were most commonly found in both the introduction/background section and the discussion/conclusion section, the same as the preretraction pairs and postretraction pairs without acknowledgment of retraction. However, when the multiple mentions were all in a single IMRaD section, the section was usually the introduction/background, and second most common was the discussion/conclusion section. Despite the slight differences, the postretraction pairs (regardless of whether the retraction was acknowledged or not) were most commonly observed in two sections: introduction/background and discussion/conclusion.

### 4.4. Intentional Postretraction Citations

#### 4.4.1. Lack of acknowledgment of retraction shown in the citation contexts

To understand, at scale, whether postretraction citations acknowledge retractions, we used the methods described in Section 3.3 to identify postretraction citation contexts acknowledging that retracted papers were cited. We identified 722 postretraction citation contexts. Note that the 722 contexts only account for 5.4% of the 13,252 postretraction citation contexts we studied. In other words, an overwhelming majority (94.6%) of the postretraction citation contexts do not show awareness of the retraction when citing retracted papers.

#### 4.4.2. Purposes for intentionally citing retracted papers

Table 9 presents the number of intentional citations we classified with each citation purpose. The annotation was done primarily by the first author. To estimate the intercoder agreement, we randomly sampled 100 citation contexts from the 722 citation contexts and assigned the sampled contexts to a graduate student in scientometrics who was not involved in developing the classification scheme. The Kappa coefficient of the intercoder agreement is .63, showing a fair agreement between the two coders (Cohen, 1960). The annotation manual was deposited to the Illinois Data Bank (Hsiao & Schneider, 2021), and a confusion matrix of the 100 annotations can be found as Supplementary material 4. Following an initial annotation, we collapsed two categories that were close in purpose because they could not be reliably distinguished. After collapsing the two categories, the main points of confusion were that the second annotator annotated all instances of the first annotator’s *Notify retraction included* as *Related work*, and interpreted *Example of problematic science* differently than the first annotator.

**Table 9.** Distribution of the citation contexts belonging to the purposes

Purpose	# citation contexts	(%)
Related work	453	(62.74)
Example of problematic science	62	(8.59)
Reproduce	40	(5.54)
Exclusion rationale	35	(4.85)
Subject of study	33	(4.57)
Comparison^a^	26	(3.60)
Notify retraction included	24	(3.32)
Use	20	(2.77)
Other	14	(1.94)
Correction	10	(1.39)
Republication of retraction	5	(0.69)
Total	722	(100)

^a^ Comparison: 11 (1.52%) were negative (−), 5 (0.69%) were neutral (±), and 10 (1.39%) were positive (+).

As shown in Table 9, the most prevalent purpose, *related work*, accounted for 453 (62.74%) of the 722 citation contexts. This provides insight into why retraction did not stop citations: Findings reported in the retracted papers were still regarded as parts of the development of a particular research topic even though some of the retracted papers were mentioned negatively. The following examples illustrate this phenomenon.

An example of a negative mention (from PMID: 17474991):

Another trial of a multivitamin and multimineral supplement in healthy elderly subjects reported beneficial effects after one year in six of seven tests [Retraction PMID: 11527656], though these findings have recently been retracted in the light of concerns about the veracity of the data and possible conflicting commercial interest [Retraction notice PMID: 11527656].

An example of a nonnegative mention (from PMID: 26029167):

One of the first biomarkers proposed was serum IGF-I. Despite the retraction of one study suggesting that elevated pre-treatment free IGF-I levels were associated with NSCLC patient response to figitumumab ([Retraction PMID: 21102589]), additional evidence supporting these findings has been published.

Furthermore, some of the *related work* citations suggest that sometimes citations to retractions might be inevitable. In these citation contexts, retracted papers were cited because the reported findings caused an important event in the field, or the reported findings were once regarded as landmarks in the field. For example, Andrew Wakefield et al.’s (1998) study (partly retracted in 2004; fully retracted in *Lancet* (2010)) on the relationship between the MMR vaccine and autism influenced the antivaccine movement. *Related work* indicates that the retracted paper(s) was cited to provide context about a background event.

The second most prevalent motive, *example of problematic science*, shows another reason why retraction did not stop citations: Retracted papers were cited to discuss problems in scientific research or scientific publishing. For instance, retracted papers were cited to provide examples of irreproducible research, scientific misconduct, and fraudulent science. We also observed cases in which retracted papers were cited to demonstrate that peer review could fail due to the fact that problematic research had been published.

**Figure 7.**
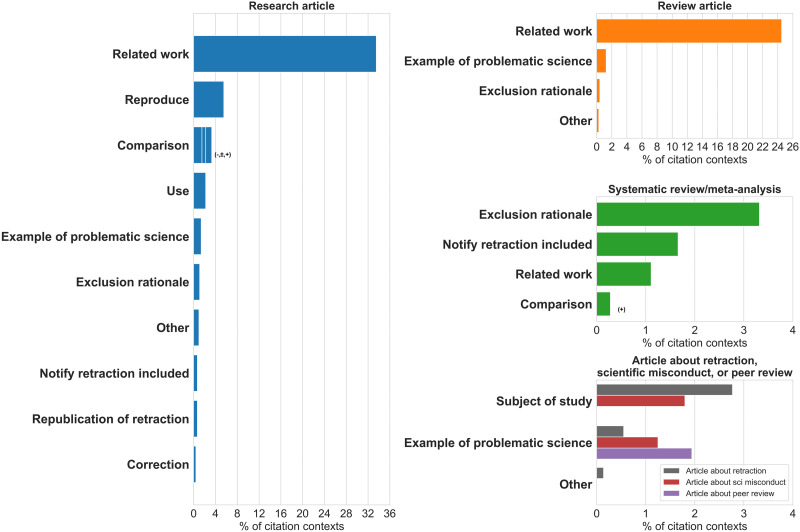
Citation purposes observed in different types of articles. The signs (−, +, ±) represent the tone identified in *comparison* citations. In research articles and review articles, most of the tones of *comparison* citations were negative (−), followed by positive (+) and neutral (±). In systematic reviews and meta-analyses, only positive *comparison* citations were observed. For articles about retraction, scientific misconduct, or peer review, the citation purposes observed in each article subtype are presented with different color keys.

While examining the citation contexts, we observed certain types of purposes in different types of citing articles (see Figure 7). We identified the types of articles in two ways. First, we collected the publication types from the metadata in the XML files. Then, we identified a set of specific article types (Supplementary material 5) and updated the types of articles for the papers that fell into these types based on specific identification rules.

Figure 7 presents the most prevalent types of articles and the citation purposes observed in the 722 citation contexts acknowledging the retraction. We found 91.14% (658) of the citation contexts in these four types of articles.

Different purposes for intentionally citing retracted papers were observed in different types of articles. *Related work* was the most common purpose observed in research articles and review articles, but not in other types of articles. In systematic reviews and meta-analyses, *exclusion rationale* was the most common purpose. This type of citation is hard to avoid, because when researchers perform systematic reviews and meta-analyses, they need to perform thorough literature searches, report the search results, and provide explicit reasons for excluding papers from the analysis. In articles about retraction or scientific misconduct, *subject of study* was the most common purpose. This type of citation is also hard to avoid because retracted papers were the “data” in these articles.

## 5. DISCUSSION AND CONCLUSIONS

Our findings show that retracted papers in biomedicine were mostly retracted within 3 years after publication and continued to be cited after retraction. Although our findings cannot be projected into the future because journals and authors can reach back in time to retract articles (e.g., PMID:1233443, retracted 45 years after publication), our findings are aligned with those of previous studies. Bar-Ilan and Halevi (2018) studied 995 retracted papers in ScienceDirect and reported that 75% of the papers were retracted no more than 3 years after publication. Chen et al. (2013) reported that, for 1,721 retracted papers indexed in WoS, the mean time to retraction was 2.57 years. Bar-Ilan and Halevi (2018) reported that postretraction citations increased; however, the growth rates of postretraction citations dropped across their three data collection dates. Dinh et al. (2019) and Mott et al. (2019) reported that retracted papers’ citation counts dropped after the retraction. Our longitudinal analysis on the shares of *active* retracted papers in each retraction year across the citation years answers RQ1 and provides further insight into the postretraction decrease in citation counts: Old retracted papers have stopped being cited as time progressed.

To answer RQ2 and RQ3, we analyzed how retracted papers were cited in full-text articles. For RQ2, the similar distributions of text progression of preretraction and postretraction citation contexts imply that the purpose for citing retraction papers did not change much before or after retraction. Interestingly, Bordignon (2020) reported that retracted/corrected papers did not receive more negative citations than nonretracted/corrected papers. Bordignon’s (2020) finding echoes ours in that retracted papers are not cited differently before and after the retraction. As for RQ3, our textual analysis of the postretraction citation contexts shows that only a limited proportion (5.4%) of postretraction citation contexts acknowledged the retraction. A similar proportion (4.15%) was reported in Budd et al.’s (2016) study covering citations to papers retracted between 2001–2005. Low proportions of postretraction citations acknowledging the retraction were also reported in some case studies (Schneider et al., 2020; van der Vet & Nijveen, 2016) and older studies (Budd et al., 1999; Pfeifer & Snodgrass, 1990). However, publicity of a retracted paper may influence the proportion of citations acknowledging the retraction. Studies on high-profile retracted papers reported higher proportions of citations acknowledging the retractions (Bornemann-Cimenti et al., 2016; Piller, 2021; Suelzer et al., 2019). Suelzer et al. (2019) studied citations to the Wakefield paper and reported that 71.7% of the citations documented the retraction after the paper was fully retracted in 2010. Bornemann-Cimenti et al. (2016) reported that 25.8% of 267 citations to 25 retracted papers by Scott S. Reuben documented the retractions. In Piller (2021), 47.5% of the citations to two retracted Covid-19 papers documented the retractions. Mott et al. (2019) also observed the difference between retracted papers with and without public attention. They reported that retracted papers that were part of large-scale retractions that received broad media attention had a larger reduction in postretraction citations than other retracted papers (Mott et al., 2019).

To answer RQ4, we analyzed the purposes for intentionally citing retracted papers. Our findings on the 722 citation contexts acknowledging retraction show that retracted papers were intentionally cited for various reasons. *Related work* was the most common purpose observed. The prevalence of *related work* partly explains why retraction did not stop citation: Findings reported in retracted papers were still regarded as part of the development of a particular research topic and might be cited to provide background context. Note that we also observed citation purposes indicating that some citations to retracted work might be inevitable, such as *exclusion rationale* and *subject of study*. Moreover, the purposes *notify retraction included* and *example of problematic science* show that citations to retracted papers can also be used for pointing out problematic science.

In summary, this study examined the postretraction citations to PubMed-indexed retracted papers. To the best of our knowledge, Budd et al.’s (2016) study covering 4,917 postretraction citations to 265 papers retracted from 2001–2005 was the most recent study on acknowledgement of retraction at scale in the biomedical field. By contrast, our study is more than twice the scale: Our study covered 13,252 postretraction citation contexts that were from 9,122 citing papers. Our findings update the understanding of postretraction citations to a vast collection of retracted papers and provide further information about how retracted papers were cited over time. This is the first large-scale study examining the citation contexts citing retracted papers. Our analysis of the locations of the citation contexts showed no significant difference in how retracted papers were cited in full-text articles before and after their retraction.

Our analysis of the purposes for intentionally citing retracted papers contributes to a deeper understanding of why retraction has not stopped citations. We are not against citing retracted papers if retracted papers are appropriately cited. The problem is that the vast majority (94.6%) of postretraction citations did not document the retraction. Previous studies (Bar-Ilan & Halevi, 2018; Schneider et al., 2020) reported that the retraction notice did not always appear when a retracted paper was searched. Often articles are not clearly labelled as retracted: This was the case for two studies of Medline-indexed papers, where 52/233 (22%) retracted papers in one sample (Decullier, Huot et al., 2013) and 15/123 (12.2%) retracted papers in a second sample (this excluded withdrawn articles) (Decullier & Maisonneuve, 2018) were not watermarked or clearly labeled. Suelzer, Deal et al. (2021) investigated how retraction information was displayed on publisher websites and six bibliographic databases (PubMed, Ovid MEDLINE, EBSCO CINAHL, ProQuest PsycINFO, Scopus, and WoS). On publisher websites, 132/150 (88%) retracted papers’ PDFs were labeled, and 109/148 (73.6%) retracted papers had links to retraction notices (Suelzer et al., 2021). Among the six databases, PubMed and Ovid MEDLINE had the best performance (150/150 (100%) labeled as retracted; 147/150 (98.0%) linked to their retraction notices). EBSCO CINAHL had the worst performance: None of the retracted papers were labeled or linked to their retraction notices (Suelzer et al., 2021). This is in contrast to the 2019 guidelines from the Committee on Publication Ethics (COPE) and the International Committee of Medical Journal Editors (ICMJE). Both the 2019 COPE and ICMJE guidelines suggest that retraction notices should be linked to the retracted paper in all online versions (Committee on Publication Ethics, Barbour et al., 2019; International Committee of Medical Journal Editors, 2019). However, resources such as Retraction Watch (2018), Zotero (2019), and scite (2020) (both of which use Retraction Watch data as of the current writing) can help authors identify retracted papers. To stop the improper spread of retracted papers, it is crucial for authors to check the retraction status and cite retracted papers carefully. At least, authors should follow guidance on citing retracted papers provided by citation standards. Guidance on citing retracted papers has been provided in popular referencing styles such as APA style (American Psychological Association, 2020), AMA style (AMA Manual of Style Committee, 2020), and NLM style (Patrias, 2007).

## ACKNOWLEDGMENTS

We thank Dr Yuanxi Fu and Malik Salami for helping us annotate the citation motives. Thanks to Dr Yuanxi Fu and Randi Proescholdt for collaborating on Table 1 for the unpublished RISRS Report, Jodi Schneider, Nathan D. Woods, Randi Proescholdt, Yuanxi Fu, and the RISRS Team: “Recommendations from the *Reducing the Inadvertent Spread of Retracted Science: Shaping a Research and Implementation Agenda* Project” MetaArXiv Preprints July 2021 (https://doi.org/10.31222/osf.io/ms579). iCite was used for public data.

## AUTHOR CONTRIBUTIONS

Tzu-Kun Hsiao: Conceptualization, Data curation, Formal analysis, Investigation, Methodology, Visualization, Writing—original draft, Writing—review & editing. Jodi Schneider: Conceptualization, Data curation, Funding acquisition, Methodology, Project administration, Supervision, Writing—review & editing.

## COMPETING INTERESTS

Tzu-Kun Hsiao has no competing interests. Jodi Schneider has been an invited speaker for the publisher organization CrossRef and has received data-in-kind from Retraction Watch and scite.

## FUNDING INFORMATION

Alfred P. Sloan Foundation G-2020-12623. NIH 5R01LM010817.

## DATA AVAILABILITY

For the data used for this study, see the Illinois Data Bank: https://doi.org/10.13012/B2IDB-8255619_V2.

## Notes

^1^ Two lead researchers (Y. Sato and J. Iwamoto) in a research group in the osteoporosis field.^2^ 
https://icite.od.nih.gov/api
^3^ The extraction of citation context is limited to PMC open access articles because this is the largest set of publicly available articles indexed in PubMed with standardized XML format that can be processed systematically.^4^ These 47 retracted papers can be found in *PubMed_retracted_publication_full_v3.tsv* (included in our dataset deposit (Hsiao & Schneider, 2021)) by filtering the *retracted_yr* column to show the blank values.^5^ Citation contexts are found concentrated at the beginning of papers the most, followed by the end of papers, for nonretracted papers as well (Bertin et al., 2016).
