# Supplementary material for: Continued use of retracted papers: Temporal trends in citations and (lack of) awareness of retractions shown in citation contexts in biomedicine
Source: Quant Sci Stud. 2022 Feb 4;2(4):1144–69. doi: 10.1162/qss_a_00155 (PMC9520488; doi:10.1162/qss_a_00155)
Supplement: Supplementary file 1 [file qss-2-4-1144-s001.pdf]

**Supplementary Materials**

Supplementary material 1. Retraction notices indexed as retracted papers

| PMID | Title | PMID of the retracted paper |
|---|---|---|
| 28756930 | Retraction notice to: Effect of transplantation of BMMSCs on pathological change of gastric precancerous lesions of rats [Asian Pac J Trop Med 8(12) (2015) 1060-1063] | 26706680 |
| 28756931 | Retraction notice to: Effect of thioredoxin-interacting protein on Wnt/β-catenin signaling pathway and diabetic myocardial infarction [Asian Pac J Trop Med 8(11) (2015) 976-982] | 26615000 |
| 31377112 | Retraction notice to "Cocrystals of curcumin-isonicotinamide and curcumin-gallic acid: Does the weak forces in cocrystals effect on binding profiles with BSA and cell cytotoxicity?" [Eur. J. Pharm. Biopharm. 140 (2019) 78-90] | 31085313 |
| 32709401 | Removal notice to: Anterior mediastinal cholesterol granuloma - A case report | 32386654 |

Supplementary material 2. Decision map

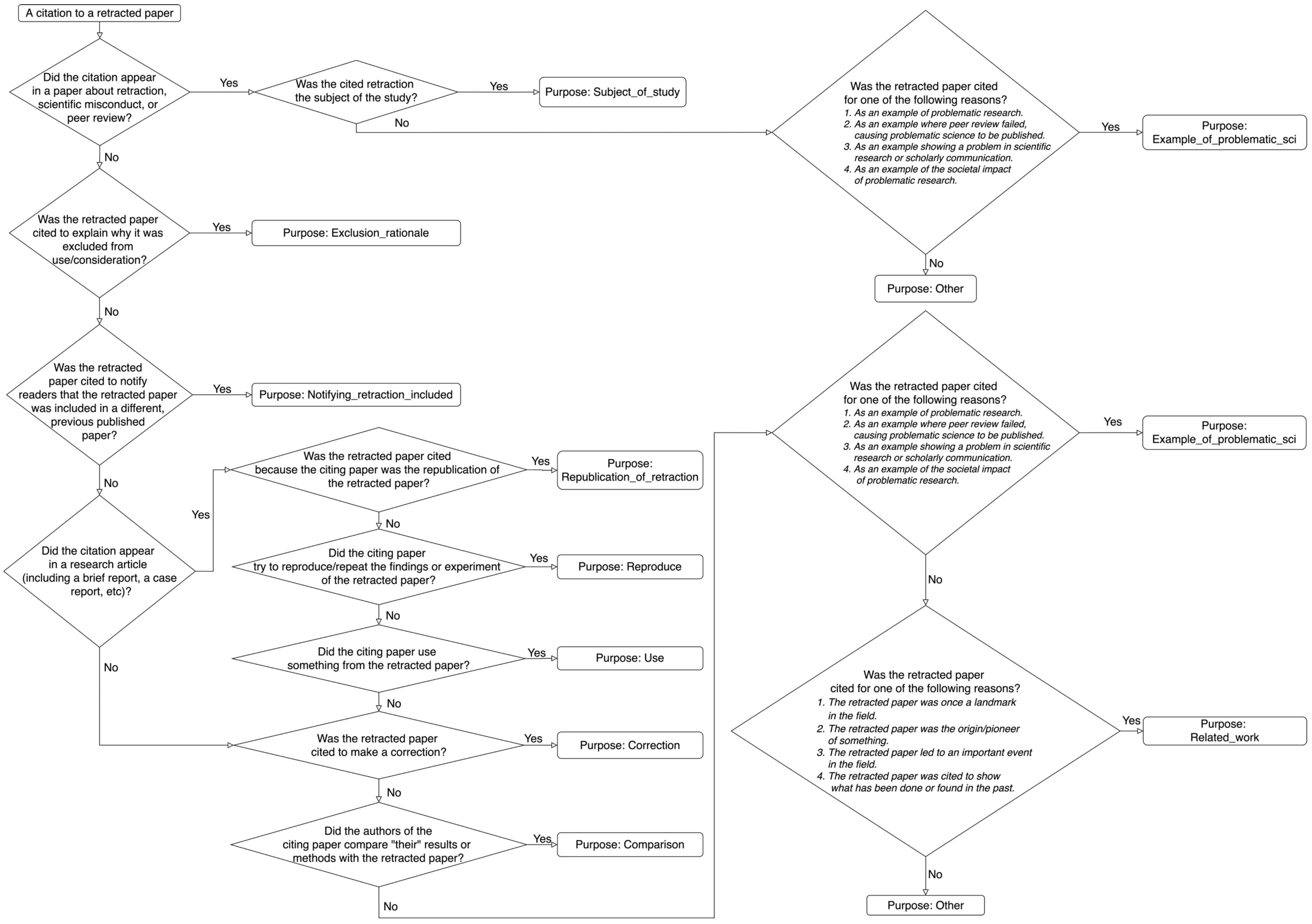

Supplementary material 3. Citation purposes and examples

| Purpose | Description | Example |
|---|---|---|
| Comparison | Authors of the citing paper compared "their" results or methods with the retracted paper. According to the tone, this category is further divided into negative (-), positive (+), and neutral (±). The negative tone refers to the cases that inconsistency, contradiction, or discrepancy is reported in the comparison. The positive tone refers to the cases that consistency is reported in the comparison. The neutral tone refers to the cases that the consistency between the compared results was unclear. | Comparison (-)<br>Overall, our results provide no evidence for a beneficial effect of multivitamin and multimineral supplementation on cognitive function in the majority of men and women 65 years and over living in the community. This result is consistent with all previous studies in non-selected elderly populations [2-10] apart from the retracted Canadian study [Retraction PMID:11527656].<br>Comparison (+)<br>Overall our findings were robust to sensitivity, subgroup, meta-regression, and trial sequential analyses (tables 3 and 4, fig 4, fig 6, and online supplement). For the comparison of albumin with control fluids, improved precision (95% confidence interval of 0.85 to 1.00; P=0.06) was observed after exclusion of studies at high risk of bias using a less appropriate fixed effects model that does not account for clinical heterogeneity. However, a definite signal of harm with albumin was not observed, consistent with large multicentre studies [Retraction PMID: 15209896].<br>Comparison (=)<br>Many secreted proteins have N-terminal leader sequences, which are cleaved during export. We have shown through silver staining and Western blot analyses of Xoo supernatants that the size of Ax21 secreted from PX099 corresponds to the size of the mature, processed protein. MS/MS analysis of the excised band reveals that peptides corresponding to Ax21 lack the N-terminal region. These results indicate that the N-terminal region of Ax21 is cleaved before or during secretion ([Retraction PMID: 19892983]). |
| Correction | The retracted paper was cited to make a correction. | In our previous publication [1], Figure 4 involved the analysis of chemotherapy-response signatures (as carried out independently by author AP and described in a 2006 Nature Medicine article [Retraction PMID: 17057710]). It has recently been determined that the chemotherapy-response signatures in [Retraction PMID: 17057710] are not reproducible, causing retraction of that article. As such, the results presented in Figure 4 of our original paper [1] are no longer valid. |
| Example of problematic science | The retracted paper was cited to provide an example of problematic science. This purpose satisfies one of the following conditions: (1) The retracted paper was cited to provide an example of problematic research (e.g., irreproducible research, unreliable research, research involving scientific misconduct, a flawed study, etc.). (2) The retracted paper was cited to provide an example where peer review failed, and problematic science was published. (3) The retracted paper was cited to provide an example showing a problem in scientific research or scholarly communication. (4) The retracted paper was cited to provide an example of the societal impact of problematic research. | There are also cases of data manipulation/fraud in NIH-supported, peer-reviewed research—more than 30 cases documented in the past 3 years according to the U.S. Public Health Service′s Office of Research Integrity (2009). One example is the study of Arnold et al. [Retraction PMID: 8633243] in which the authors reported huge synergistic effects of endocrine disruptors in the yeast estrogen assay in vitro. McLachlan [Retraction notice PMID:9254413] rescinded that paper because neither his laboratory nor others could replicate the findings. It was later determined that there was scientific misconduct and the original data were fabricated (NIH 2001). |
| Exclusion rationale | The retracted paper was cited to explain why it is excluded from use/consideration. Especially found in the context of research synthesis (e.g. review articles and meta-analyses which provide a formal exclusion rationale for papers that are not included.) This purpose can also be found in the literature review section of a research article. | The meta-analysis presented here provides a valid and up to date summary of the relevant literature, including a recently published randomised controlled trial of 339 patients with a confirmed diagnosis of uncomplicated appendicitis. It excludes the study that has been retracted subsequent to publication [Retraction PMID: 19277796], as well as another for which it was not clear if patients were randomised. |

| Purpose | Description | Example |
| --- | --- | --- |
| Notify retraction included | Notify readers that one or more retracted papers were included in a different, previous published review article, guideline, or paper. | Systematic reviews and meta-analyses of the trials, including a Cochrane review comparing antibiotic treatment and appendicectomy, published in recent years summarised the evidence as either in favour of antibiotic treatment or inconclusive. This could possibly result from inclusion of trials with poor methods or retracted since publication [Retraction PMID: 19277796], or from simplifying the evidence as a summary of both randomised and non-randomised studies. |
| Related work | The retracted paper was cited to show what has been done or found in the past or was cited for one of the following reasons: (1) The Retracted paper was once a landmark in the field; (2) the retracted paper was the origin/pioneer of something (e.g., "X first identified/describe Y", "X was identified as a novel ....", "X was initially proposed...", or "X was originally..."); (3) the retracted paper led to an important event in the field, such as Wakefield's paper's influence on autism-vaccine link and the anti-vaccine movement. | The COOPERATE study [Retraction PMID: 12531578] even showed that dual therapy with trandolapril and losartan reduced the risk of the primary endpoint (time to doubling of serum creatinine level or end stage renal disease) by 60% better than monotherapy, thereby becoming one of the most widely quoted studies by the Lancet. After such seemingly robust evidence many physicians accepted that reduction of albuminuria or proteinuria was synonymous with nephroprotection.<br>On the other hand, some researchers have asserted that acupuncture point stimulation caused human somatosensory cortex responses being detected in an fMRI. This phenomenon has been regarded as indirect evidence of the specificity of acupuncture points, although the evidences are debatable ([Retraction PMID: 9482945]).<br>Despite the retraction of one study suggesting that elevated pre-treatment free IGF-I levels were associated with NSCLC patient response to figitumumab ([Retraction PMID: 21102589]), additional evidence supporting these findings has been published.<br>As the main type of ovarian cancer, ovarian epithelial carcinoma accounts for 85% to 90% of all ovarian cancers. Moreover, the majority (65%–75%) of women with ovarian cancer are diagnosed with advanced stage disease (III and IV), and only about 15% to 20% of these women are free of disease recurrence at 10 years [Retraction PMID: 25544369]. |
| Republication of retraction | In the republication of the retracted paper, the authors cited the retracted paper to announce the republication. | This article is a revised version of a paper of the same title [Retraction PMID: 22241970] that was previously published in PLOS Computational Biology and was subsequently retracted when a computational error was discovered. |
| Reproduce | A citation to the retracted paper was made because the citing paper tried to reproduce/repeat the finding or experiment mentioned in the retracted paper. | In a further effort to obtain an intelligence priming effect, we attempted to replicate Gordijn and Stapel's study. Their article [Retraction PMID: 17393877] was retracted after the completion of Experiment 7 and hence their data can be given no evidential weight. Nevertheless, the hypothesis they put forward is a reasonable one and thus we report Experiment 7 in relation to that hypothesis. |
| Subject of study | Cited retraction is the object of study of a case study about retraction, or is the data used in a study about retraction, scientific misconduct, or peer review. Note that in these studies, retracted papers can be cited in the results. | Our objective was to test whether such simple tools applied to a manuscript known to be fraudulent [Retraction PMID: 19923501] would have helped to detect some warning signals of poor quality. |
| Use | Citing paper uses something from the cited retracted paper. This type of citation is often found in Methods section. | To verify whether the above-mentioned points have the potential of playing substantial roles in explaining this story, we planned a proof-of-concept survey of DNA sequence databases, and namely: (i) A bioinformatic analysis on the primer sequences described by Lombardi et al ([Retraction PMID: 19815723]), compared against human and murine (house mouse Mus musculus) genomes. |
| Other | Those do not belong to the above categories. | -- |

Supplementary material 4. Confusion matrix of the 100 annotations of citation purpose

| | | Annotator 1 | | | | | | | | | | | |
|---|---|---|---|---|---|---|---|---|---|---|---|---|---|
| Annotator 2 | | Comparison | Correction | Example of problematic science | Exclusion rationale | Notify retraction included | Other | Related work | Reproduce | Republication of retraction | Subject of study | Use | Total |
| | Comparison | 4 | 0 | 0 | 0 | 0 | 0 | 1 | 0 | 0 | 0 | 0 | 5 |
| | Correction | 0 | 0 | 0 | 0 | 0 | 0 | 1 | 0 | 0 | 0 | 0 | 1 |
| | Example of problematic science | 0 | 0 | 3 | 0 | 0 | 0 | 3 | 0 | 0 | 0 | 0 | 6 |
| | Exclusion rationale | 0 | 0 | 0 | 9 | 0 | 0 | 0 | 0 | 0 | 0 | 0 | 9 |
| | Notify retraction included | 0 | 0 | 0 | 1 | 0 | 0 | 0 | 0 | 0 | 0 | 0 | 1 |
| | Other | 0 | 0 | 2 | 0 | 0 | 0 | 0 | 0 | 0 | 1 | 0 | 3 |
| | Related work | 0 | 0 | 1 | 0 | 6 | 0 | 64 | 0 | 0 | 0 | 0 | 71 |
| | Reproduce | 0 | 0 | 0 | 0 | 0 | 0 | 1 | 0 | 0 | 0 | 0 | 1 |
| | Republication of retraction | 0 | 0 | 0 | 0 | 0 | 0 | 0 | 0 | 0 | 0 | 0 | 0 |
| | Subject of study | 0 | 0 | 0 | 0 | 0 | 0 | 0 | 0 | 0 | 1 | 0 | 1 |
| | Use | 0 | 0 | 0 | 0 | 0 | 0 | 1 | 0 | 0 | 0 | 1 | 2 |
| | Total | 4 | 0 | 6 | 10 | 6 | 0 | 71 | 0 | 0 | 2 | 1 | 100 |

Supplementary material 5. Rules for identifying papers with specific types

| Type | Priority | Identification rule |
|---|---|---|
| Retraction notice | 1 | Papers queried from PubMed using *"Retraction of Publication"[pt]*. |
| Erratum | 2 | Papers queried from PubMed using *"Published Erratum"[pt]*. |
| Article about retraction | 3 | Papers queried from PubMed using *"Retraction of Publication as Topic" [mh]* or title contains *retract** or *withdr**. |
| Article about peer review | 4 | Title contains *peer review*. |
| Article about scientific misconduct | 5 | Title contains at least one of the following words: *misconduct, plagiar*, fraud, reproducibility, forgery, unreliable research,* and *image duplication*. |
| Systematic review/ meta-analysis | 6 | Title contains *systematic review* or *meta-analysis*. |
